# Association of radiotherapy with thoracic vertebral fractures in esophageal squamous cell carcinoma: A retrospective cohort study

**DOI:** 10.1097/MD.0000000000035304

**Published:** 2023-09-29

**Authors:** Xing-Qiang Wu, Tian-Yue Zhang, Fan Yang, Xin-Yi Feng, Yu-Ling Feng, Ling-Li Wang, Tian-Wu Chen, Chun-Ping Li, Rui Li

**Affiliations:** a Department of Radiology, Affiliated Hospital of North Sichuan Medical College, and Sichuan Key Laboratory of Medical Imaging, Sichuan, China; b Department of Radiology, The second Affiliated Hospital of Chongqing Medical University, Chongqing, China.

**Keywords:** esophageal squamous cell carcinoma, radiotherapy, vertebral fracture

## Abstract

To investigate the association between radiotherapy (RT) and thoracic vertebral fractures in esophageal squamous cell carcinoma (ESCC) and explore the risk factors of thoracic vertebral fracture in ESCC who underwent RT. This retrospective cohort study including 602 consecutive ESCC patients examined the association between RT and thoracic vertebral fractures using multivariable Cox proportional hazard models and relevant risk factors of thoracic vertebral fractures based on clinical and RT parameters in patients with ESCC. Followed for a median follow-up of 24 months, 54 patients had thoracic vertebral fractures. The multivariable analysis revealed RT as an independent risk factor after adjusting for clinical risk factors. Univariable analyses associated a 5-Gy increase in vertebral dose to single vertebrae and a 1-time increase in RT fraction with higher risk of vertebral fracture. Adding RT factors (vertebral dose and fraction) and mean vertebral hounsfield unit to the Cox models containing conventional clinical risk factors significantly improved the χ^2^ value for predicting vertebral fractures (all *P* < .001). This study revealed RT, as well as increased vertebral dose and RT fractions, as a significant, consistent, and strong vertebral fracture predictor in ESCC. Combined vertebral dose, RT fractions, and vertebral hounsfield unit provided optimal risk stratification for ESCC patients.

## 1. Introduction

Esophageal cancer is one of the most common digestive tumors in China. It ranks third in incidence and fourth in mortality of malignant tumors, with squamous cell carcinoma as the predominant pathological type.^[1]^ Radiotherapy (RT) is one of the most important modalities in esophageal cancer treatment, either in curative or palliative settings. Preoperative RT could reduce the tumor diameter, nodal involvement, and metastasis stages to enable surgical availability, whereas postoperative RT can significantly improve overall survival (OS). However, patients with esophageal squamous cell carcinoma (ESCC) who underwent RT may suffer some complications, including radiation pneumonia, esophagitis, and esophagobronchial or esophago-mediastinal fistula. RT can also lead to incidental thoracic vertebral irradiation due to its presence within the irradiation field, resulting in an increased risk of thoracic vertebral fractures.

In recent years, Fujii et al reported that RT was associated with thoracic vertebral fractures in esophageal cancer, correlating the risk with the mean vertebral dose.^[2]^ The widely implemented advanced RT technology, including intensity-modulated RT, stereotactic body RT, volumetric modulated arc therapy, and deep inspiration breath-hold, has reduced the vertebral dose.^[3]^ However, the optimal RT dosage for patients with ESCC remained controversial, due to no low threshold dose, in which vertebral fracture does not occur, was determined, resulting in radiation-induced vertebral damage remains potential and severe and late ESCC complications after RT. Most previous studies on radiation-induced bone damage focused on total and single doses,^[2–4]^ while the RT fraction has received less attention. Whether RT fractions play a key role in thoracic vertebral fracture development is not completely understood.

Furthermore, Fujii found that the decreased mean thoracic vertebral hounsfield unit (HU) also contributed to the higher risk of fracture. However, there is a lack of data concerning the impact of RT dose across different strata of mean HU of vertebrae and a simple risk prediction model that integrates RT status and vertebral attenuation.^[2]^ Thus, our study aims to investigate the risk factors for thoracic vertebral fracture occurrence in patients with ESCC and explore the correlation between vertebral fracture and the components of total radiation dose and fraction, as well as mean HU of thoracic vertebrae, to provide effective clinical preventive measures and improve the prognosis of patients with ESCC.

## 2. Methods

### 2.1. Patients

**Figure 1.**
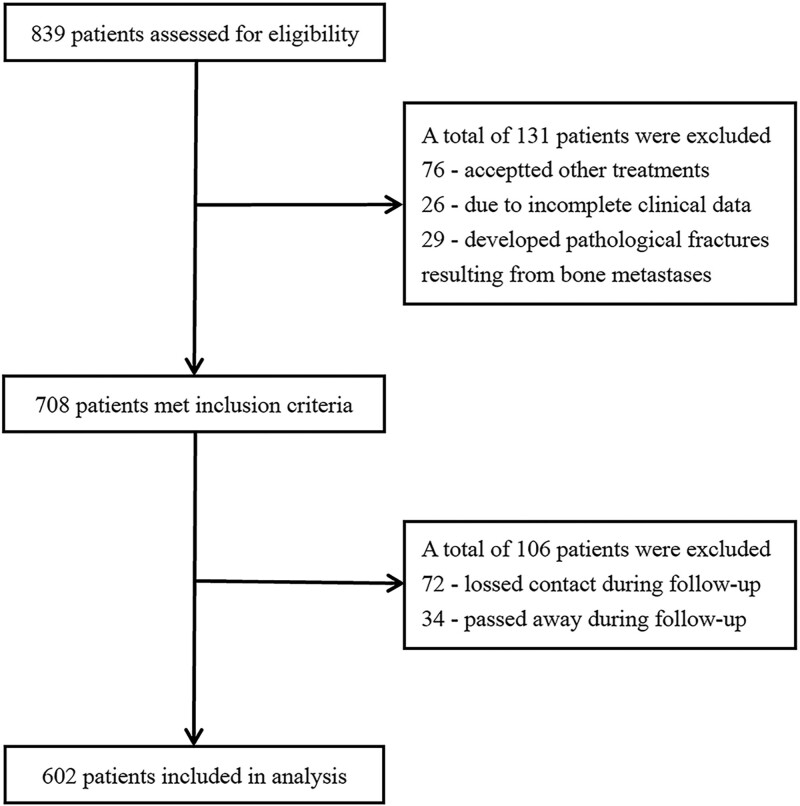
Flow chart detailing the identification of the study cohort.

The study protocol was approved by Medical Ethics Committee of Affiliated Hospital of North Sichuan Medical College in 2020 (2020ER[A]008) and conformed to the principles of the Declaration of Helsinki, and each participant provided written informed consent. All methods were carried out in accordance with relevant guidelines and regulations. From March 2016 to June 2019, 839 consecutive patients with pathologically proven ESCC were retrospectively reviewed according to the following inclusion criteria: clinical stage diagnoses according to the eighth edition of the American Joint Committee on Cancer for esophageal cancer,^[5]^ no tumor-related treatment before diagnosis, and acceptable chest computed tomography (CT) imaging quality. The exclusion criteria included: incomplete clinical data and pathological fractures resulting from bone metastases or direct tumor invasion in the vertebrae. In total, 602 patients enrolled in our study; 119 received single RT, 359 received chemoradiotherapy (CRT), and 124 did not, respectively, serving as RT, CRT, and Non-RT groups (Fig. 1).

### 2.2. CT examination

Initial CT examination was performed by Simens SOMATOM Force 128-Layer CT, GE Light Speed VCT 128-Layer CT, and Philips Brilliance 128-Layer CT. Follow-up patients were scanned with the same CT scanner as much as possible. Patients were placed supine and scanned from the root of the neck to the level of both suprarenal calyces. The CT scan includes the following parameters: tube voltage of 120 kV, tube current of 100 mA, a scan layer thickness of 5 mm, layer spacing of 5 mm, spherical tube speed of 0.5 s/r, filter function of FC10, matrix of 512 × 512, and pitch of 0.984:1. Enhancement scans were performed by injecting the nonionic isotonic contrast agent iohexol (containing 300 mg/mL of iodine) through the elbow vein via a high-pressure syringe at an injection flow rate of 3.0 to 3.5 mL/second. The raw scan data was transferred to the post-processing workstation for reconstruction to obtain a reconstructed sagittal image of the patient vertebrae, with a reconstructed layer thickness of 1 or 1.25 mm and layer spacing of 1 or 1.25 mm.

### 2.3. CT image analysis

**Figure 2.**
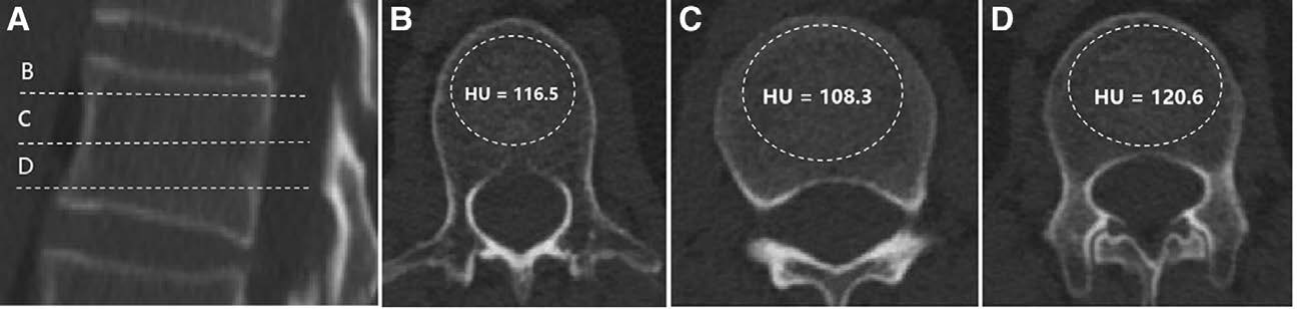
Computed tomography scans illustrating the technique for calculating vertebral bone mineral density (BMD) with HUs. (A) Sagittal slice of the thoracic vertebral body demonstrating axial planes of interest. (B–D) Axial images showing hounsfield unit (HU) values calculated by the plain computed tomography (CT) of chest.

Two radiologists (with 9 and 10 years of experience in chest imaging, respectively) independently reviewed all data and unanimously determined whether a thoracic vertebral fracture had occurred. Fracture was defined as losing at least 20% of height in the sagittal plane or 10% of the vertebral area observed on sagittal reconstructed CT images.^[6,7]^ Furthermore, the mean thoracic vertebral HU was measured on both initial and last follow-up unenhanced CT images. The region of interest was manually drawn at 3 points on axial images of the thoracic vertebral as parallel to the endplates as possible. The region of interest was drawn by encapsulating only the cancellous bone and avoiding cortical edges, osseous abnormalities, and voids, such as vascular channels (Fig. 2). The average of the 3 attenuation value measurements served as the final HU for individual vertebral levels.^[8]^ The differences between the initial and follow-up vertebral (ΔHU) attenuation values were calculated by the following formula: ΔHU = HUpre − HUpost. Intra- and inter-observer variability of HU values was obtained by comparing the measurements by the same observer in 30 random cases to verify the measurement repeatability. The measuring time interval of the same observer is 2 weeks. Inter-observer variability was accessed by another independent double-blinded observer with 3 years of experience in chest imaging.

### 2.4. Treatment details

Among 602 cases, 413 had primary tumor resection. In the RT groups, 388 received RT for primary tumor region, 67 received additional RT for non-regional lymph nodes, and 90 received RT for postoperative recurrence or metastasis. The median total radiation dose was 60.9 Gy (range: 20.0–124.1 Gy), and the median dose delivered to the thoracic vertebrae was 36.23 Gy (quantile: 26.6–41.3; range: 2.5–67.6 Gy). Most patients (75.3%, 360/478) receive conventionally fractionated RT, with a median single dose of 2.0 (1.8~2.0) Gy/f/d, 5f/w. A total of 424 patients received chemotherapy, which was performed after initial CT examination, including paclitaxel + platinum (n = 214, 50.5%), vincristine + platinum (n = 59, 13.9%), irinotecan + platinum (n = 44, 10.4%), docetaxel + platinum (n = 83, 19.6%), and 5-fluorouracil + calcium folinic acid + cisplatin (n = 24, 5.6%).

### 2.5. Follow-up

The frequency of follow-up was performed at 3-month intervals from the initial treatment in the first year and then 6-month intervals until 1 year post-treatment. Thoracic CT scans were performed at each follow-up. The presence of thoracic vertebral fractures was identified on follow-up reconstructed CT images. The follow-up duration was calculated from the first thoracic CT examination until the thoracic vertebral fracture occurrence or last CT scanning with the patient. The follow-up ended on June 30, 2021.

### 2.6. Statistical analysis

All statistical analyses were performed using the Statistical Package for the Social Sciences (version 26.0), R (version 4.1.1), and MedCalc software (version 20.02). Variables were expressed as number (%), mean ± standard deviation, or median (interquartile range, Q25–75). Categorical variables were compared by chi-square test and continuous variables by Student *t* test, Mann–Whitney *U* test, or Kruskal–Wallis *H* test. The Kappa test analyzed the consistency of RT irradiation fields and thoracic vertebral fracture location. Receiver-operating characteristic curves were built to determine the best threshold for quantitative variables to detect the vertebral fracture. The optimal cutoff point was identified using the Youden index. The coefficient of variation was used to assess the thoracic vertebral HU measurement reproducibility. The Kaplan–Meier method with a log-rank test assessed thoracic vertebral fractures between groups, stratified by the optimal cutoff point and a log-rank test. The univariate Cox proportional hazards regression model was used to test the risk factors associated between thoracic vertebral fractures and potential confounders. Subsequently, the multivariate models of Cox regression started with all variables showing a significant association with the effect of univariate Cox analysis. Clinical critical variables of age and sex were considered to enter in a forward stepwise multivariate Cox analysis. Multivariate models, including RT factors, mean vertebral HU, and their combination with clinical parameters, was created to investigate the impact of potential confounders and outcomes. The incremental values of adding RT factors and mean vertebral HU to Cox models were studied by calculating the improvement of the χ^2^ values and Harrell C-index. *P* values of < .05 were considered a statistically significant difference.

## 3. Results

### 3.1. Baseline characteristics

The final cohort comprised 602 patients, of whom 190 (31.6%) were female. The mean age was 64.01 ± 8.18 years, with 280 (46.5%) with advanced-stage diseases (stage III or IV) at initial evaluation (*P* < .001) and 354 (58.8%) with tumors located in the middle thorax (*P* = .001). Additionally, patients who underwent RT were older and were more likely females (*P* = .018). Table 1 presents the baseline information.

**Table 1:** Baseline between groups.

	Non-RT (n = 124)	CRT (n = 359)	RT (n = 119)	*P* value
Age, yr	62.41 ± 7.65	63.47 ± 7.85	67.32 ± 8.81	<.001
Female (%)	29 (23.3)	113 (31.4)	48 (40.3)	.018
BMI, kg/m^2^	20.73 ± 2.22	21.42 ± 2.51	20.54 ± 2.65	<.001
History of smoking (%)	60 (48.4)	153 (42.6)	30 (25.2)	<.001
History of alcohol excess (%)	47 (37.9)	110 (30.6)	20 (16.8)	.001
Clinical stage (%)^a^				<.001
I	2 (1.6)	5 (1.4)	1 (0.8)	
II	89 (71.8)	166 (46.2)	59 (49.6)	
III	17 (13.7)	119 (33.1)	29 (24.4)	
IV	16 (12.9)	69 (19.2)	30 (25.2)	
Tumor location (%)				.001
Upper thoracic	10 (8.1)	73 (20.3)	24 (20.2)	
Middle thoracic	71 (57.3)	208 (57.9)	75 (63.0)	
Lower thoracic	43 (34.7)	78 (21.7)	20 (16.8)	
Resection of primary esophageal tumor (%)	104 (83.9)	236 (65.7)	73 (61.3)	<.001
Chemotherapy (%)	65 (52.4)	359 (100)	-	-
Radiotherapy technique (%)				.442
IMRT	-	315 (87.7)	101 (84.9)	
VMAT	-	30 (8.4)	10 (8.4)	
3D-CRT	-	14 (3.9)	8 (6.7)	
Total dose, Gy	-	61.2 (57.2~64.8)	60.0 (54.0~64.0)	.52
Dose per fraction, Gy	-	2.0 (1.8~2.1)	2.0 (1.8~2.0)	.051
Radiotherapy fraction	-	31 (30~33)	30 (28~33)	.08
Radiotherapy course ≥2 (%)	-	30 (8.3)	13 (10.9)	.39

Variables are number (%), mean ± SD or median (interquartile range, IQR, Q_25_–Q_75_).

3D-CRT = three dimensional conformal radiation therapy, BMI = body mass index, CRT = chemoradiotherapy, IMRT = intensity modulated radiation therapy, Non-RT = non-radiotherapy, RT = radiotherapy, VMAT = volumetric modulated arc therapy.

a Defined using the eighth edition of the AJCC Cancer Staging Manual.

### 3.2. Follow-up

**Figure 3.**
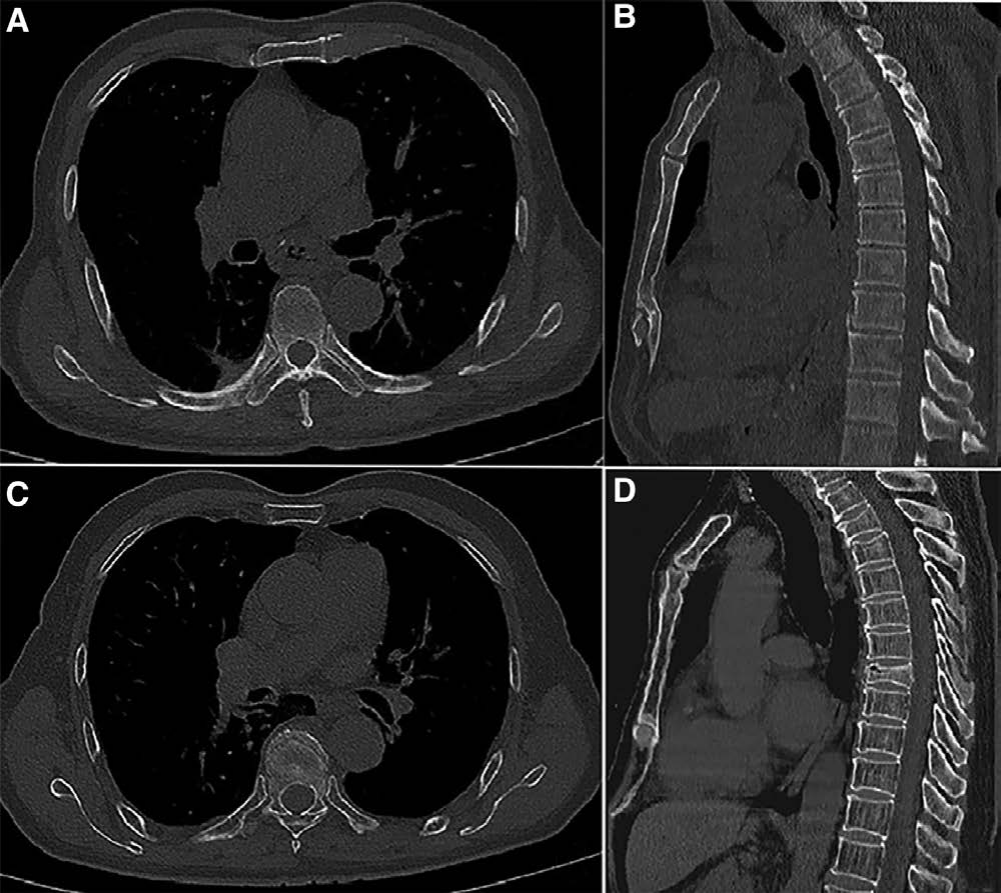
Axis and multiplanar reformation in sagittal views of computed tomography (CT) images before and after radiotherapy. (A and B) Initial CT images showing absence of thoracic vertebral fracture in Axial (A) and Sagittal planes (B) before radiotherapy. (C and D) Follow-up CT scanning showed T7 thoracic vertebral insufficiency fractures 23 mo after the radiotherapy.

During a median follow-up of 24 months (range: 4.0–51.0 months), 54 patients experienced thoracic vertebral fractures, including 49 (10.25%) who had RT and 5 (4.03%) who did not. And the median observation time of vertebral fracture after RT was 15 months (range: 12.5–25.5 months). Of 54 patients, 46 had a single vertebral fracture, whereas 8 had multiple fractures, including 7 with 2 vertebral and 1 with 3 vertebral fractures (Fig. 3). Meanwhile, in our study, the cumulative incidence rates of vertebral fractures in the 3 groups during the first year were as follows: RT group (5/119; 4.20%), CRT group (9/359; 2.51%), and Non-RT group (0/124; 0%). In the second year, the cumulative incidence rates of vertebral fractures in the 3 groups were: RT group (4/119; 3.36%), CRT group (18/359; 5.01%), and Non-RT group (1/124; 0.8%). Additionally, the mean interval from the first CT examination to the occurrence of vertebral fracture in the RT and CRT groups was lower than that in the Non-RT group (19.50 ± 8.72 and 18.20 ± 7.28 vs 26.40 ± 7.89 months, respectively); however, the differences did not reach a statistical significance (*P* = .11).

### 3.3. Intra- and inter-observer variability of HU measurements of thoracic vertebral

Thoracic HU values revealed good reproducibility of all thoracic vertebrae, with intra- and inter-observer coefficient of variation of 2.34% and 5.78%, respectively. As illustrated in Table 2, the mean CT value of thoracic vertebral decreased significantly during follow-up compared with the initial CT examination in the RT (150.63 ± 29.65 vs 117.39 ± 33.12 HU, *P* < .001), CRT (152.44 ± 25.59 vs 111.38 ± 25.61 HU, *P* < .001), and Non-RT group (157.51 ± 27.78 vs 149.05 ± 30.77 HU, *P* = .043). However, the degree of decrease in thoracic vertebral attenuation of patients in the Non-RT group was lower than that of the RT and CRT groups.

**Table 2:** Risk factors of patients with univariable and multivariable analyses.

	Univariable		Multivariable	
Variable	HR (95%CI)	*P* value	HR (95%CI)^a^	*P* value
Age (per 1 yr increase)	1.09 (1.05~1.13)	<.001	1.04 (1.01~1.08)	.037
Female	1.20 (0.69~2.10)	.52	1.43 (0.72~2.85)	.30
BMI (per 1 kg/m^2^)	0.95 (0.84~1.07)	.44	-	-
History of smoking	1.28 (0.75~2.20)	.35	-	-
History of alcohol excess	1.88 (1.09~3.23)	.022	2.73 (1.41~5.29)	.003
Clinical stage				
I	1 [Reference]	NA	-	-
II	0.43 (0.06~3.18)	.41	-	-
III	0.53 (0.07~4.02)	.54	-	-
IV	0.49 (0.06~3.90)	.50	-	-
Tumor location				
Upper thoracic	1 [Reference]	NA	-	-
Middle thoracic	1.42 (0.63~3.19)	.39	-	-
Lower thoracic	1.00 (0.38~2.58)	.99	-	-
Resection of primary esophageal tumor	0.67 (0.39~1.17)	.16	-	-
Chemotherapy	0.98 (0.56~1.75)	.96	-	-
Treatment modality				
Non-RT	1 [Reference]	NA	1 [Reference]	NA
RT	4.62 (1.65~12.96)	.004	3.58 (1.27~10.23)	.017
CRT	3.54 (1.38~9.09)	.009	3.57 (1.38~9.25)	.008
HU (Per 10HU increase)	0.63 (0.56~0.72)	<.001	0.66 (0.57~0.76)	<.001

BMI = body mass index, CI = confidence interval, CRT = chemoradiotherapy, Gy = Gray, HR = hazard ratio, HU = hounsfield unit, NA = not applicable, Non-RT = non-radiotherapy, RT = radiotherapy.

a Adjust for age, female and history of alcohol excess.

### 3.4. Risk factors for thoracic vertebral fracture

**Figure 4.**
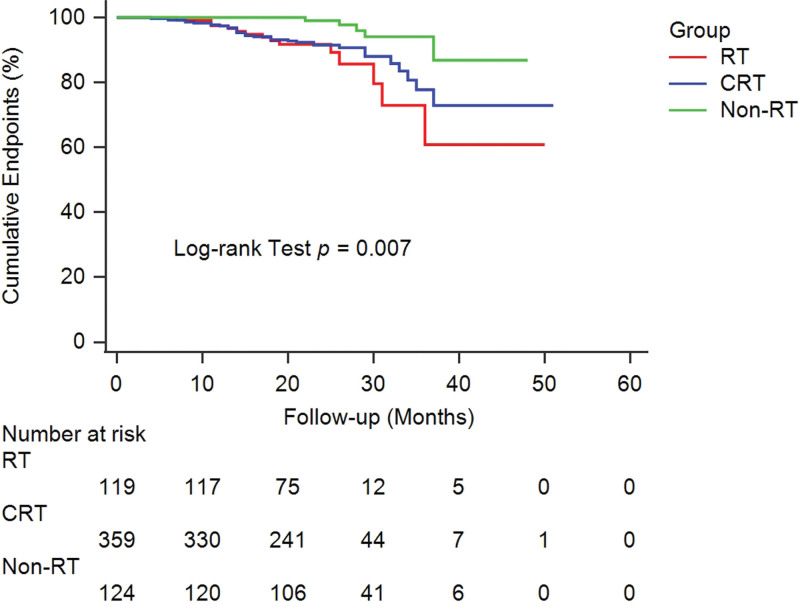
Kaplan–Meier curves examining the time to the occurrence of vertebral fracture based on the status of radiotherapy.

The univariable Cox regression analysis showed that RT (RT: hazard ratio [HR]: 4.62, 95% confidence interval [CI]: 1.65–12.96, *P* = .004; and CRT: HR: 3.54, 95% CI: 1.38–9.09, *P* = .009) (Fig. 4), age (HR: 1.09, 95% CI: 1.05–1.13, *P* < .001), and moderate alcohol excess (HR: 1.88, 95% CI: 1.09–3.23, *P* = .022) were risk factors for vertebral fractures. Additionally, patients with higher thoracic vertebral attenuation had less chance of developing thoracic vertebral fractures (10 increased mean HU: HR: 0.63, 95% CI: 0.56–0.72, *P* < .001). As illustrated by Table 2, the multivariable Cox proportional hazards analysis revealed that patients who underwent RT had an estimated HR for thoracic vertebral fracture (RT: HR: 3.58, 95% CI: 1.27–10.23, *P* = .017 and CRT: HR: 3.57, 95% CI: 1.38–9.25, *P* = .008) compared with patients who did not. However, gender, body mass index, smoking history, clinical stage, tumor location, surgery, and chemotherapy do not show visible effects on thoracic fractures (All *P* > .05).

### 3.5. Association of thoracic vertebral fracture location and RT field

The thoracic vertebral fracture location is highly consistent with the range of radiation fields in patients receiving RT (κ = 0.867, *P* < .001, Table 3). Meanwhile, the most common fracture sites of thoracic vertebrae after RT are thoracic vertebrae 7 and 8. Contrastingly, the fractured thoracic vertebrae are mainly in the lower thoracic in patients who did not receive RT, including the 9th, 11th, and 12th thoracic vertebrae, which are consistent with the vertebrae that were frequently fractured secondary to age-related osteoporosis.

**Table 3:** Consistency analysis of radiation field and thoracic fractures in radiotherapy (RT) and chemoradiotherapy (CRT) group.

Radiation field	n	Thoracic fractures	n	κ value	*P* value
Upper thoracic	7	Upper thoracic	4	0.867	<.001
Middle thoracic	33	Middle thoracic	36		
Lower thoracic	9	Lower thoracic	9		
Total	49		49		

### 3.6. Incremental prognostic value of adding RT factors and vertebral HU values in predicting thoracic vertebral fractures

**Figure 5.**
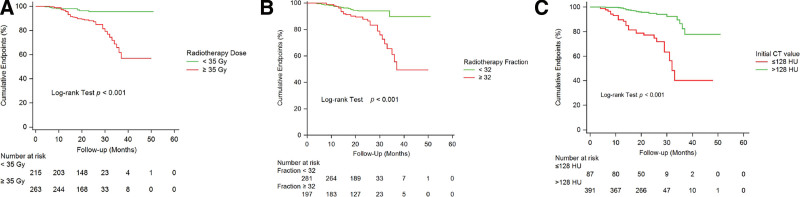
Kaplan–Meier curves examining the time to the occurrence of vertebral fracture based on the cutoff value of (A) radiotherapy dose, (B) radiotherapy fraction, and (C) the initial computed tomography (CT) value of thoracic vertebrae.

The receiver-operating characteristic curve analysis showed that the optimal values of vertebral dose, fractions, and vertebral HU values for identifying patients with vertebral fracture were 35 Gy (area under the curve [AUC] = 0.637), 32 fractions (AUC = 0.687), and 128 HU (AUC = 0.756), respectively. The Kaplan–Meier curves analysis revealed that stratified vertebral dose and RT fractions by cutoff values, high-dose delivered to the thoracic vertebrae (>35 Gy), and hyper-fractions (>32 times) groups had a greater risk of thoracic vertebral fracture (χ^2^ = 15.74, log-rank: *P* < .001, Fig. 5A, and χ^2^ = 14.06, log-rank: *P* < .001, Fig. 5B). For the initial CT values, the risk of thoracic vertebral fracture increased significantly when the thoracic vertebral HU decreased to approximately 128 HU (χ^2^ = 46.33, log-rank: *P* < .001, Fig. 5C).

**Figure 6.**
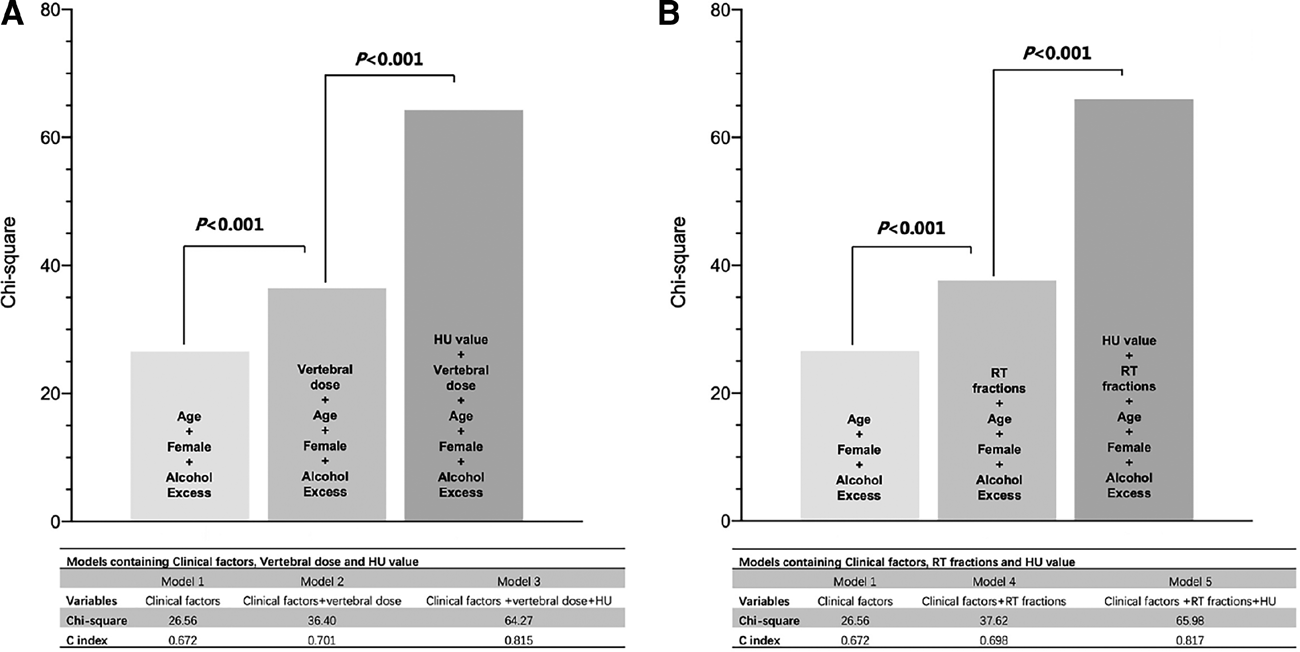
Incremental prognostic value of radiotherapy (RT) factors and vertebral hounsfield unit (HU) value over conventional clinical characteristics to occurrence of vertebral fracture. The comparison between models (*P* values) was conducted by χ^2^ test.

The univariable Cox regression analyses indicated that age (HR: 1.07, 95% CI: 1.04–1.11), moderate alcohol excess (HR: 1.89, 95% CI: 1.07–3.36), 10 HU increase of vertebral HU (HR: 0.65, 95% CI: 0.57–0.74), 5 Gy increase of vertebral dose (HR: 1.22, 95% CI: 1.09–1.36), a one-time increase of RT fractions (HR: 1.05, 95% CI: 1.02–1.08), and RT course (HR: 2.53; 95% CI: 1.30–4.94) have significant associations with the thoracic vertebral fractures (Table 4). Thus, various Cox models investigated the significance of RT factors and the mean vertebral HU in predicting endpoints. The multivariate stepwise analyses results revealed that adding RT factors (vertebral dose and fractions) and mean vertebral HU to the model containing conventional clinical factors (age, gender, and history of alcohol excess) had significantly improved models for predicting thoracic vertebral fractures with higher χ^2^ value and C-index (All *P* < .001), (Table 4, Fig. 6). No strong collinearity was found (all VIF < 1.5) among the covariates in the multivariable models.

**Table 4:** Results of univariable and multivariable analyses for radiotherapy (RT) patients.

	HR (95% CI)	*P* value	C-index
Univariable			
Age (per 1 yr increase)	1.07 (1.04~1.11)	<.001	0.645
Female	1.11 (0.62~1.99)	.72	0.501
BMI (per 1 kg/m^2^)	0.88 (0.78~1.01)	.07	
History of smoking	1.41 (0.81~2.48)	.22	
History of alcohol excess	1.89 (1.07~3.36)	.028	0.536
Clinical stage	0.99 (0.69~1.43)	.98	
Tumor location	1.08 (0.69~1.68)	.73	
Resection of primary esophageal tumor	0.84 (0.4~1.47)	.55	
Radiotherapy modality	0.59 (0.25~1.39)	.23	
Radiotherapy dose (per 5 Gy increase)	1.22 (1.09~1.36)	<.001	0.551
Dose per fraction	1.83 (0.22~15.3)	.58	
Radiotherapy fractions	1.05 (1.02~1.08)	<.001	0.596
Radiotherapy course ≥2	2.53 (1.30~4.94)	.006	0.514
HU (Per 10 HU increase)	0.65 (0.57~0.74)	<.001	0.736
Multivariable			
Model 1			
Age (per 1 yr increase)	1.08 (1.04~1.12)	<.001	0.672
Female	1.77 (0.87~3.61)	.11	
History of alcohol excess	2.88 (1.43~5.82)	.003	
Model 2			
Age (per 1 yr increase)	1.08 (1.04~1.12)	<.001	0.701
Female	1.68 (0.84~3.33)	.14	
History of alcohol excess	2.64 (1.34~5.22)	.005	
Radiotherapy dose (per 5 Gy increase)	1.10 (1.04~1.18)	.001	
Model 3			
Age (per 1 yr increase)	1.04 (1.00~1.08)	.038	0.815
Female	1.37 (0.69~2.73)	.36	
History of alcohol excess	2.60 (1.32~5.12)	.006	
HU (Per 10HU increase)	0.69 (0.59~0.79)	<.001	
Radiotherapy dose (per 5 Gy increase)	1.17 (1.04~1.31)	.005	
Model 4			
Age (per 1 yr increase)	1.08 (1.04~1.12)	<.001	0.698
Female	1.68 (0.84~3.33)	.14	
History of alcohol excess	2.60 (1.31~5.15)	.006	
Radiotherapy fractions	1.04 (1.02~1.07)	<.001	
Model 5			
Age (per 1 yr increase)	1.04 (0.99~1.08)	.057	0.817
Female	1.39 (0.70~2.72)	.35	
History of alcohol excess	2.51 (1.28~4.93)	.008	
HU (Per 10HU increase)	0.69 (0.59~0.79)	<.001	

BMI = body mass index, CI = confidence interval, Gy = Gray, HR = hazard ratio, HU = hounsfield unit.

## 4. Discussion

The current study examines the association between RT and the risk of thoracic vertebral fractures in patients with ESCC. The results associated RT with an increased risk of thoracic vertebral insufficiency fractures, which remained an independent risk factor after comprehensive adjustment for age, gender, moderate alcohol excess, and initial CT value of vertebrae. The vertebral fracture incidence increased with more RT fractions and higher vertebral doses, which were not reported in previous studies. Third, models that incorporate the clinical, RT, and CT parameters could better help determine whether patients with ESCC who receive RT may have a greater risk of thoracic vertebral fracture.

Previous studies confirmed that RT may cause fractures in several malignant tumors, including gastric cancer, non-small cell lung carcinoma, rectal cancer, and cervical cancer, which reduced vertebra bone mineral density (BMD) after RT and with a higher incidence of vertebral and pelvic fractures.^[9–14]^ In our study, patients with ESCC who underwent RT had a higher risk of thoracic vertebral fracture (10.25% vs 4.03%), consistent with previous studies,^[2,10,14]^ indicating that thoracic vertebral fracture was not a rare adverse event after RT in ESCC and should be emphasized in the clinical setting.

Patients with ESCC who received a higher RT dose had a greater risk of vertebral fracture because the radiation dose to the thoracic vertebrae increased with the total RT dose. The pathophysiological mechanism of bone injury, including high-dose x-rays, could directly destroy the small vessels and capillary injury of vertebrae, leading to osteoclast, osteoblast cell death, and fatty infiltration into the bone marrow, eventually causing BMD loss, skeletal architecture deterioration, and even vertebral fractures.^[15–17]^ However, the optimal RT dose for esophageal cancer was uncertain. The recent meta-analysis by Sun and Xiao et al^[18,19]^ revealed that high-dose RT, especially ≥60 Gy, could improve the OS and local-regional control in esophageal cancer without increasing severe complications compared with low-dose RT. Thus, advanced RT technology that allowed a marked vertebral dose reduction should be considered in further study.

Moreover, we further assess the association between vertebral dose (single and total), and RT fractions with the risk of vertebral fractures and investigate the factor that may have a greater effect on vertebral fractures. The present study confirmed that total vertebral dose and RT fractions were strong independent vertebral fracture predictors after adjusting for known risk factors, including moderate alcohol excess, age, and female gender. The combination of RT dose and fractions could provide better vertebral fracture risk stratification in patients with ESCC.

In addition to patients underwent more RT fraction may receive a higher total dose, another potential mechanism of higher fractions impacting the risk of vertebral fracture in ESCC might there has no uncommonly accepted dose tolerance for bone injury, which is likely a non-stochastic effect with no dose threshold and no minimum risk threshold.^[20]^

There is no consensus on radiation regimen and fractionation protocols for specific patients, however, hypo-fractionated RT allows for higher doses per fraction, with modern precision RT, are associated with a more effective biologically effective dose, local control and OS rate, and less lung toxicity.^[21–24]^ Patients with ESCC may also benefit from hypo-fractionated RT for lower vertebral fracture risk and the advantages mentioned above. However, this hypo-fractionated radiation modality needs to be confirmed by further prospective studies, which also need to pay more attention to the dose per fraction. Moreover, the risk of thoracic vertebral fracture after RT for ESCC may decrease with the advanced RT guidance techniques, which deliver a higher dose to the tumor accompanied by minimal damage to the adjacent organ, such as the thoracic vertebrae.^[25]^

Moreover, most thoracic vertebral fractures occurred in the radiation field in this cohort, which was contrary to the thoracic vertebral fractures caused by age-related osteoporosis and usually observed in the lower thoracic vertebrae. This finding illustrated a strongly positive correlation between fracture location and radiation field (*k* = 0.867, *P* < .001). Meanwhile, the mean vertebral HU (reflecting the BMD) was also significantly decreased in the radiation region compared to the initial CT examination, suggesting that radiation could cause damage to adjacent bones after RT. However, patients who underwent RT did not suffer from fractures. Additionally, patients associated with a higher initial CT value of thoracic vertebral after RT had less vertebral fracture incidence (HR: 0.63, 95% CI: 0.56–0.72, *P* < .001). HU value inclusion resulted in Cox models that could significantly increase the power and accuracy of models for predicting vertebral fracture. The utility of CT scan in providing reliable estimates for BMD in ESCC should also be considered because CT has been extensively applied for preoperative diagnosis, therapeutic efficacy, and prognostic assessment of esophageal tumors.^[26]^ However, advanced RT modalities should be performed to reduce the vertebral dose and decrease RT fractions, if possible, in patients with lower initial CT values. During follow-up, more sensitive imaging, such as vertebral magnetic resonance, should be considered in patients with a high risk of vertebral fracture to monitor the edema and unnoticed vertebral structure abnormalities, which were not visible on conventional CT examination.

## 5. Limitations

This study had the following limitations. Firstly, this retrospective analysis was a single-center study, which may affect the analysis results. Therefore, future research should expand upon and validate the presented results by a larger, multi-center prospective trial. Secondly, a small percentage of patients were not scanned with the same scanner and sequence during the follow-up period, which may have little influence on the thoracic vertebral HU measurement. Thirdly, as total dose vary among ESCC patients in this cohort, the impact of increasing RT fractions on the risk of vertebral fracture in this study may be related to higher total radiation dose. Therefore, the potential influence of RT fractions on fracture risk at the similar level of total dose needs to be further explored. Finally, we estimated when thoracic vertebral fractures appeared as first seen on the follow-up CT. However, fractures would have occurred within the intervals from the previous follow-up CT, which may underestimate the duration of such events.

## 6. Conclusion

RT was associated with an increased risk of vertebral fracture in ESCC in this study. Mean vertebral dose, RT fractions, and initial vertebral HU were independent prognostic values for vertebral fracture with clinical risk factors. A vertebral dose, RT fractions, and vertebral HU provided optimal risk stratification for patients with ESCC.

## Author contributions

**Conceptualization:** Xing-Qiang Wu, Tian-Yue Zhang, Fan Yang, Xin-Yi Feng, Yu-Ling Feng, Rui Li.

**Data curation:** Ling-Li Wang.

**Project administration:** Rui Li.

**Supervision:** Fan Yang, Tian-Wu Chen, Chun-Ping Li, Rui Li.

**Writing – original draft:** Xing-Qiang Wu, Tian-Yue Zhang.

**Writing – review & editing:** Xing-Qiang Wu, Tian-Yue Zhang, Tian-Wu Chen, Chun-Ping Li.
